# Lateral Pancreaticojejunostomy in a Rare Case of Chronic Calcific Pancreatitis With Peripancreatic Pseudocyst in an 18-Year-Old Male: A Case Report

**DOI:** 10.7759/cureus.59843

**Published:** 2024-05-07

**Authors:** Sandeep Reddy Ramala, Suresh R Chandak, Souvik Sarkar, Raju K Shinde, Meenakshi S Chandak

**Affiliations:** 1 General Surgery, Jawaharlal Nehru Medical College, Datta Meghe Institute of Higher Education and Research, Wardha, IND; 2 Respiratory Medicine, Jawaharlal Nehru Medical College, Datta Meghe Institute of Higher Education and Research, Wardha, IND; 3 Dermatology, Jawaharlal Nehru Medical College, Datta Meghe Institute of Higher Education and Research, Wardha, IND

**Keywords:** magnetic resonance cholangiopancreatography (mrcp), lateral pancreatico jejunostomy, abdominal ultrasonography, pseudocyst of the pancreas, chronic calcific pancreatitis

## Abstract

Another name for the Puestow surgery is a lateral pancreaticojejunostomy. The primary pancreatic duct, which runs from the head to the tail of the organ, is opened, exposing the pancreas. In order to allow the pancreas to empty straight into the intestines, the opening of the pancreatic duct is subsequently joined to a tiny intestinal loop. For more than 50 years, this process has been used to effectively relieve pain caused by chronic pancreatitis. This technique has a very low mortality rate and a low rate of surgical complications, and a high success rate. The gradual fibrosis of the pancreas resulting in the loss of exocrine and endocrine function is known as chronic pancreatitis. Intense pain is the disease's most typical symptom. It is unclear what causes the discomfort in chronic pancreatitis. Nonetheless, a large number of these patients have dilated ducts that are made up of intervening structures and saccular dilations, a condition known as the "chain of lakes" phenomenon. Radiological investigations can be used for diagnosis in these patients. Lateral pancreaticojejunostomy is the most effective treatment option for these individuals. Preservation of endocrine and exocrine pancreatic function is another benefit of lateral pancreaticojejunostomy. With lateral pancreaticojejunostomy, chronic fibrocalcific pancreatitis that manifests as pancreatic ductal dilatation and persistent discomfort can be effectively treated. Excellent early outcomes have been observed in terms of pain alleviation as well as post-operative morbidity and mortality; however, the patient's overall outcome and long-term follow-up have not been as well defined.

## Introduction

Loss of endocrine and exocrine function as well as increasing pancreatic fibrosis are hallmarks of chronic pancreatitis. Pain is the most typical sign of chronic pancreatitis, and in certain cases, it can be excruciating and unmanageable [1]. Patients who have developed complications like intestinal obstruction, calcific pancreatitis, pleural effusion, and pseudocyst, as well as those who have continuous pain that is not responsive to medical therapy, must have surgery [2]. It's unclear what causes discomfort in chronic pancreatitis. It frequently comes before any exocrine or endocrine function loss as well as any radiographically visible pancreatic alterations. According to certain data [3], pain may be caused by perineural inflammation. An obstruction-related dilated pancreatic duct may raise intraductal pressures and produce pain [4]. Achieving primary pain relief and improving quality of life are the main goals of therapy. This could be accomplished by endotherapy, surgery, or other forms of care [5]. Fibrosis causes the pancreas to first harden and expand. The most frequent cause of chronic pancreatitis is alcohol consumption. Familial and idiopathic groups, however, are also widely acknowledged. Tropical calcific pancreatitis (TCP) is frequent in several tropical locations [6]. The medical treatment of this illness entails prescribing pancreatic enzyme supplements as well as managing diabetes and discomfort. An effective peripancreatic pseudocyst around the tail and chronic calcific pancreatitis in an 18-year-old male patient was treated with a lateral pancreatojejunostomy.

## Case presentation

An 18-year-old male presented with complaints of episodes of pain in the abdomen for 2.5 years to the surgery outpatient department of a tertiary care hospital. Pain in the abdomen, which was sudden and insidious in onset, had increased in the past two months and was continuous. Pain from the epigastric region spread to the back, accompanied by nausea that was made worse by eating and only somewhat better by analgesics, which resulted in loss of appetite and loss of weight. There was no family history of similar conditions and no history of any addictions. The patient was taking pancreatic enzyme supplements and analgesics after being admitted for similar concerns in the past. Physical examination and abdominal examination were normal. Blood investigations such as complete blood count (CBC), liver function test (LFT), kidney function test (KFT), international normalised ratio (INR), amylase, and lipase values were within normal limits. Chest x-ray and ECG findings were normal.

USG of the abdomen and pelvis showed mild atrophy of the pancreatic body and tail region. Intraductal calculus was seen in the region of the pancreatic head 6.2mm with dilatation of the pancreatic duct. Smaller calculi were also seen in the pancreatic duct in the region of the body of pancreas. A small cystic lesion was seen in the pancreatic tail region measuring 2.4 x 1.6cm.

**Figure 1:**
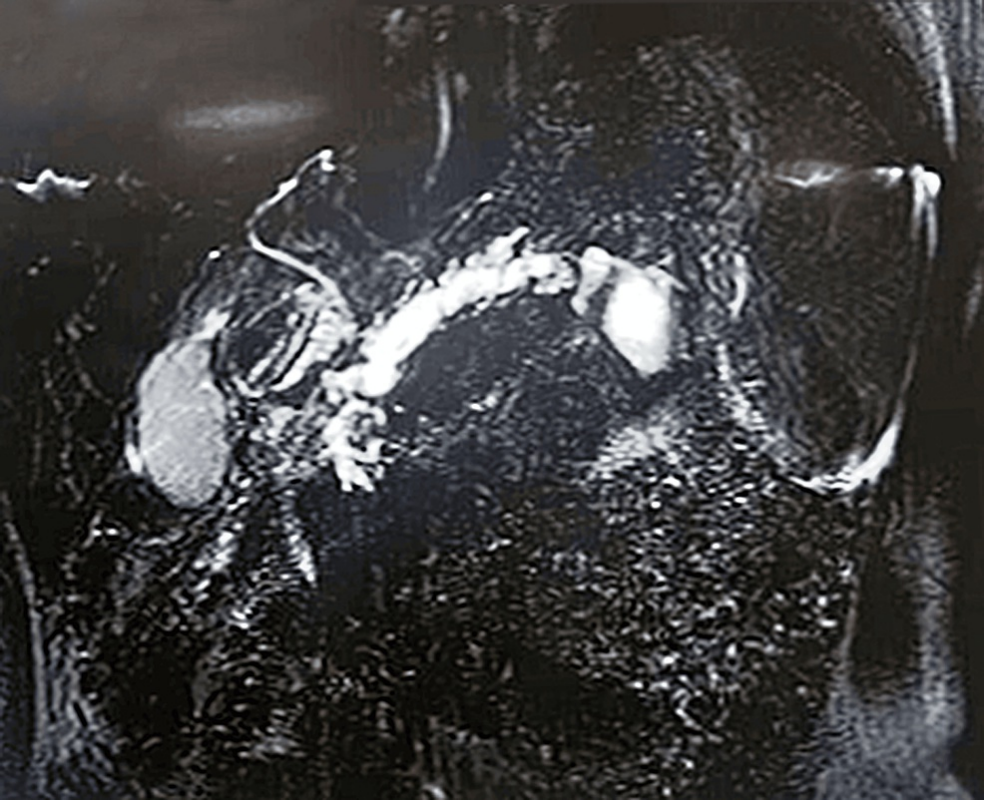
MR cholangiopancreatography image showing dilated pancreatic duct with intraductal calculi in the neck

MR cholangiopancreatography (MRCP) showed moderate diffuse pancreatic parenchymal atrophy, with significant dilatation of the main pancreatic duct as well as its side branches and focal intermittent luminal narrowings (giving beaded appearance to the pancreatic ductal system). The maximum diameter of the dilated pancreatic duct measured approximately 9-10 mm. Few intraluminal filling defects, suggesting intraductal calculi, were seen in the dilated pancreatic duct in the neck and head regions - the largest such calculus measured 8 x 5 mm. Multiple small parenchymal calcifications were seen throughout the pancreas. A thin-walled cystic lesion, measuring 2. 3 x 2 by 1.8 cm, was seen just inferior to the tail of the pancreas (peripancreatic pseudocyst) (Figure 1). 

Preoperatively, the patient was advised to have a high-protein diet and chest physiotherapy. Under general anaesthesia and epidural anaesthesia, lateral pancreaticojejunostomy was done. The rooftop incision was deepened in layers, peritoneum opened and visceral organs were identified.

**Figure 2:**
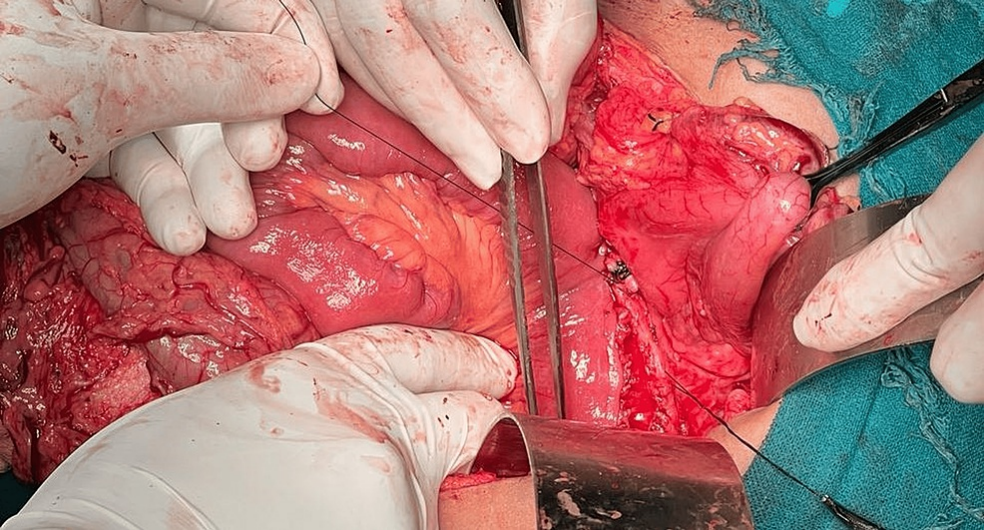
Intraoperative picture of lateral pancreaticojejunostomy

**Figure 3:**
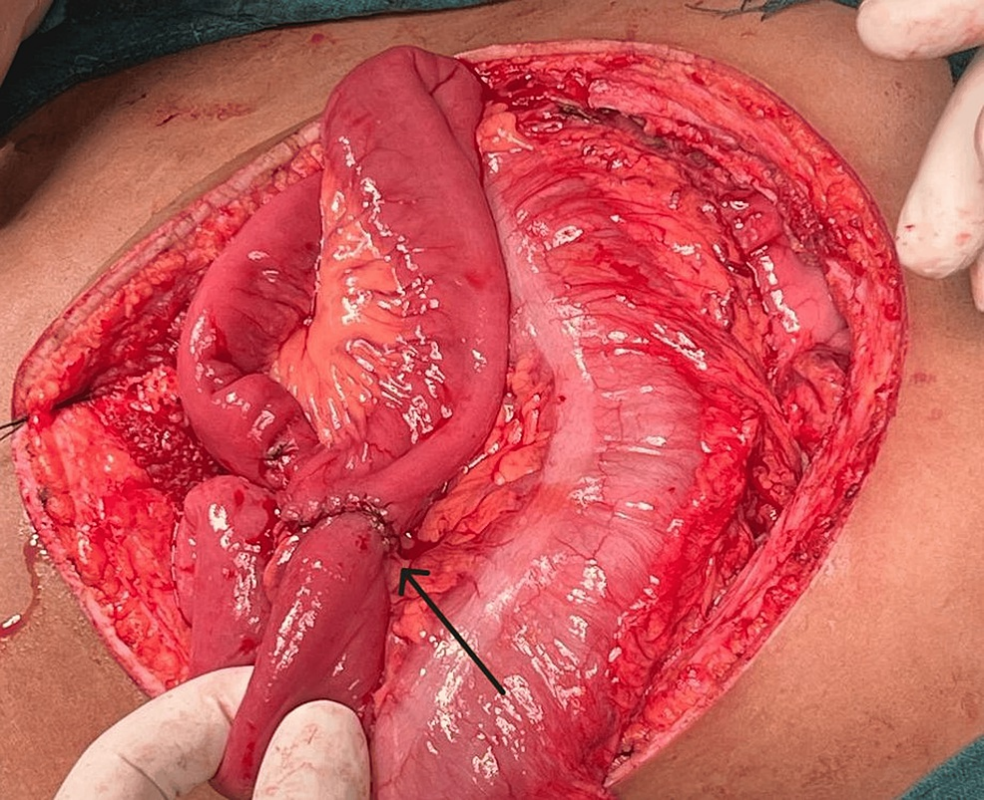
Intraoperative picture of jejunojejunostomy (black arrow)

Kocherisation of the duodenum was done and the pancreas was identified with a pseudocyst in the tail of the pancreas. The pancreas was separated from the stomach, duodenum, and transverse colon. Confirmation of the main pancreatic duct (MPD) was done by aspiration with a syringe. After confirmation, MPD was opened longitudinally. Multiple small calculi were noted within the pancreatic duct. These calculi were removed. Jejunal loop was taken around 40-50 cm from the duodenojejunal (DJ) junction. Lateral pancreaticojejunostomy was done. Bilateral abdominal drain no. 24 was placed. The closure of layers was done and the patient was shifted to the ICU post-operatively (Figures 2, 3). 

**Figure 4:**
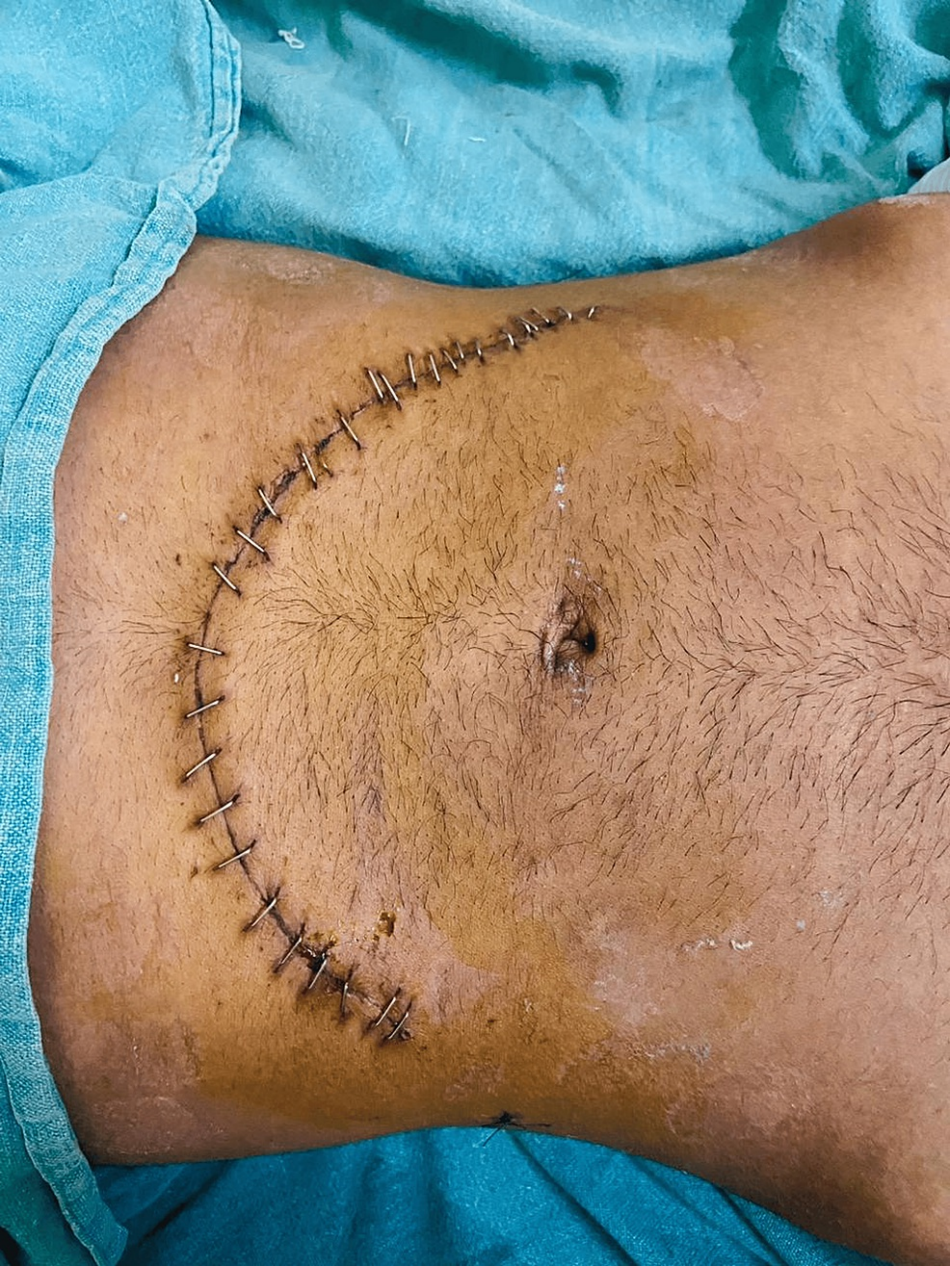
Postoperative picture showing a healthy scar line.

Post-operatively, the patient was managed with IV fluids, injectable antibiotics, injectable analgesics, and injectable antiemetics. On post-operative day 1, the patient was mobilized with an abdominal binder, and chest physiotherapy and nebulization were given. On post-operative day 5, abdominal drain was removed and a check dress was done. The suture line was healthy, with no gaps and no discharge (Figure 4).

Post-operatively, the patient recovered well and was discharged.

## Discussion

A continuing inflammatory condition of the pancreas that results in permanent morphological abnormalities is called chronic pancreatitis. Although inherited and idiopathic groups are also well-known, drunkenness is the only known cause of chronic pancreatitis in the Western world. Tropical calcific pancreatitis is a term used to describe chronic pancreatitis with stone formation and duct strictures that occur at a significantly younger age in some tropical nations [6]. According to data from a national epidemiological survey carried out in Japan, people with chronic pancreatitis had a pancreatic cancer risk that was almost 11.8 times higher than that of the general population [7]. The treatments for chronic pancreatitis have a variety of goals, the main ones being pain relief and reducing the chance of developing more serious issues, such as pancreatic cancer [8,9]. It has been demonstrated that pancreaticojejunostomy surgery, which modifies the pancreatic duct drainage to address the underlying cause of inflammation, can stop chronic pancreatitis from worsening [10].

Some people are diagnosed with idiopathic chronic pancreatitis because they experience recurrent episodes of pancreatitis for an unidentified or undetermined aetiology. The term "tropical calcific pancreatitis" (TCP) was initially used in a report by Zuidema [11] among young diabetics in Indonesia, where he noted pancreatic calcification and fibrosis [12]. The development of TCP has been linked to genetic aetiologies, including mutations in the serine protease inhibitor Kazal type 1 (*SPINK1*) gene [13]. TCP is strongly correlated with changes in the genes *SPINK1* and *Chymotrypsinogen C*. Mutations in transcription factor 7-like 2, glycoprotein 2, cathepsin B, carboxypeptidase A1, cystic fibrosis transmembrane conductance regulator (CFTR), and the calcium-sensing receptor gene are among the others [12]. A number of factors, including genetics, oxidative stress, free radical damage, trace element insufficiency, ingestion of cassava (cyanogen toxicity), and starvation, all contribute to the pathogenesis, but the exact cause of the condition is still unknown [12]. Patients are finally diagnosed with idiopathic chronic pancreatitis (ICP) when they do not fit the criteria for tropical calcific pancreatitis. Therefore, it is critical that clinicians assess the patients presenting with signs and symptoms of chronic pancreatitis when there is no evidence of gallstones, ethanol, toxic exposure, or family history for the diagnosis of ICP or TCP. These individuals should be recommended imaging tests in addition to the clinicians performing a thorough evaluation of their pancreatic function to determine the extent of pancreatic damage and pancreatic calcification.

## Conclusions

For excruciating obstructive chronic pancreatitis, surgery is the most effective treatment, according to new high-quality evidence. Inflammatory pancreatitis known as tropical calcific pancreatitis is typically linked to pancreatic duct stones, for which lateral pancreaticojejunostomy yields good outcomes with manageable early morbidity and death. Although the risk of pancreatic cancer is decreased by surgical treatments for chronic pancreatitis, it should be remembered that pancreatic cancer can still develop years after surgery.
